# Supplementary material for: JIP4 deficiency causes a lysosomal storage disease arising from impaired cystine efflux
Source: bioRxiv. 2025 Jun 8:2025.06.06.657909. Preprint. [Version 1] doi: 10.1101/2025.06.06.657909 (PMC12258935; doi:10.1101/2025.06.06.657909)
Supplement: 1 [file NIHPP2025.06.06.657909v1-supplement-1.pdf]

bioRxiv preprint doi: https://doi.org/10.1101/2025.06.06.657909; this version posted June 9, 2025. The copyright holder for this preprint (which was not certified by peer review) is the author/funder, who has granted bioRxiv a license to display the preprint in perpetuity. It is made available under aCC-BY 4.0 International license.

Supplemental Data for:

JIP4 deficiency causes a lysosomal storage disease arising from impaired cystine efflux

Layla M. Nassar[1,2,8], Xiaojian Shi[4,6,7], Agnes Roczniak-Ferguson[1,2,8], Hongying Shen[4,6,7], and Shawn M. Ferguson[1,2,3,4,5,8*]

Departments of Cell Biology[1], Department of Neuroscience[2], Program in Cellular Neuroscience, Neurodegeneration and Repair[3], Wu Tsai Institute[4], Kavli Institute for Neuroscience[5], Department of Cellular and Molecular Physiology[6], Yale University School of Medicine, New Haven, Connecticut 06510, USA. Systems Biology Institute[7], Yale West Campus, West Haven, CT 06516, USA. Aligning Science Across Parkinson's (ASAP) Collaborative Research Network, Chevy Chase, MD, 20815, USA.[8]

*Correspondence: shawn.ferguson@yale.edu

bioRxiv preprint doi: https://doi.org/10.1101/2025.06.06.657909; this version posted June 9, 2025. The copyright holder for this preprint (which was not certified by peer review) is the author/funder, who has granted bioRxiv a license to display the preprint in perpetuity. It is made available under aCC-BY 4.0 International license.

Table S1: Summary of antibodies used in this study

Antibody	Acquired from	Reference number	Concentration
FLAG	Cell Signaling Technologies	2368, RRID:AB_2217020)	WB: 1:5000<br>IF: 1:100
JIP4	Cell Signaling Technologies	5519, RRID:AB_10828724	WB: 1:2500<br>IF: 1:100
GAPDH	Encor Biotechnology	MCA-1D4, RRID:AB_2107599	WB: 1:60000
HA	Cell Signaling Technologies	3724S, RRID:AB_1549585	WB: 1:1000
Vinculin	Cell Signaling Technologies	13901, RRID:AB_2728768	WB: 1:10000
LC3B	MBL International	PM036, RRID:AB_2274121	WB: 1:1000
LAMP1 (H4A3)	DSHB	H4A3, RRID:AB_2296838	WB: 1:10000<br>IF: 1:1000
TMEM55B	Proteintech	23992-1-AP, RRID:AB_2879391	WB: 1:5000
NEDD4	Cell Signaling Technologies	2740, RRID:AB_2149312	WB: 1:5000
RFP	Rockland	600-401-379, RRID:AB_2209751	WB: 1:10000
LAMP1 (D2D11)	DSHB	9091S, RRID:AB_2687579)	WB: 1:10000<br>IF: 1:1000
anti-megalin/LRP2	Gift from Dr Daniel Biemsderfer and Dr Peter Aronson; Yale University	MC-220 , RRID:AB_3696948	WB: 1:1000<br>IF: 1:100
Cathepsin D	R&D Systems	MAB1029, RRID:AB_2292411	WB: 1:5000<br>IF: 1:100
Clathrin Light Chain	EMD Millipore / Sigma	AB9884, RRID:AB_11211734	WB: 1:1000<br>IF: 1:100
Rat IgG (HRP)	Cell Signaling Technologies	7077S, RRID:AB_10694715	1:2000
Rabbit IgG (HRP)	Cell Signaling Technologies	7074S, RRID:AB_2099233	1:2000
Mouse IgG (HRP)	Cell Signaling Technologies	7076S, RRID:AB_330924	1:2000
AlexaFluor 488 anti-rat	Invitrogen	A21208, RRID:AB_2535794	1:200
Alexa 488-Rabbit	Invitrogen	A21206, RRID:AB_2535792	1:200
Alexa 568-Rabbit	Invitrogen	A10042, RRID:AB_2534017	1:200
Alexa 647-Mouse	Invitrogen	A21202, RRID:AB_141607	1:200

bioRxiv preprint doi: https://doi.org/10.1101/2025.06.06.657909; this version posted June 9, 2025. The copyright holder for this preprint (which was not certified by peer review) is the author/funder, who has granted bioRxiv a license to display the preprint in perpetuity. It is made available under aCC-BY 4.0 International license.

Alexa 594-Goat	Invitrogen	A11058, RRID:AB_2534105	1:200

Table S2: Summary of primers oligonucleotide sequences and crRNA used in this study

Construct	Sequence (5'->3')	Reference	Notes
JIP4 gRNA 1 F	CACCGACGAGAAAATCCAGCCATGC	This paper	Forward primer for generating JIP4 gRNA construct 1
JIP4 gRNA 1 R	AAACGCATGGCTGGATTTTCTCGTC	This paper	Reverse primer for generating JIP4 gRNA construct 1
JIP4 gRNA 2 F	CACCGTTGCAGCACATCTTTCTCAC	This paper	Forward primer for generating JIP4 gRNA construct 2
JIP4 gRNA 2 R	AAACGTGAGAAAGATGTGCTGCAAC	This paper	Reverse primer for generating JIP4 gRNA construct 2
pLVX-JIP4-FLAG F	TGCAGTCGACGGTACCGCGGGCCCGCCACCATGGAGCTGGAGGACGG	This paper	Forward primer for ligating JIP4-FLAG into pLVX backbone
pLVX-JIP4-FLAG R	TCTAGAGTCGCGGGATCCCTTATCACTTATCGTCGTCATCC	This paper	Forward primer for ligating JIP4-FLAG into pLVX backbone
CTNS K190A K329A F	TTGGAGGCGGGCAAGTGTCATTG	This paper	Forward primer for generating K->A mutants at K190A and K329A for CTNS-RUSH-K->A and pLVX-CTNS-FLAG-K->A constructs
CTNS K190A K329A R	CCCTCAGTGCTTGCGTAGTAAAAG	This paper	Reverse primer for generating K->A mutants at K190A and K329A for CTNS-RUSH-K->A and pLVX-CTNS-FLAG-K->A constructs
CTNS K329A K396A F	CTTTTACTACGCAAGCACTGAGGG	This paper	Forward primer for generating K->A mutants at K329A and K396A for CTNS-RUSH-K->A and pLVX-CTNS-FLAG-K->A constructs
CTNS K329A K396A R	CATACCCCGGTCTCGCTCTGTACAAA	This paper	Reverse primer for generating K->A mutants at K329A and K396A for CTNS-RUSH-K->A and pLVX-CTNS-FLAG-K->A constructs
CTNS K396A K190A F	TTTGTACAGAGCGAGACCGGGGTA	This paper	Forward primer for generating K->A mutants at K396A and

bioRxiv preprint doi: https://doi.org/10.1101/2025.06.06.657909; this version posted June 9, 2025. The copyright holder for this preprint (which was not certified by peer review) is the author/funder, who has granted bioRxiv a license to display the preprint in perpetuity. It is made available under aCC-BY 4.0 International license.

			K190A for CTNS-RUSH-K->A and pLVX-CTNS-FLAG-K->A constructs
CTNS K396A K190A R	CAATGACACTTG CCCGCCTCCAA	This paper	Reverse primer for generating K->A mutants at K396A and K190A for CTNS-RUSH-K->A and pLVX-CTNS-FLAG-K->A constructs
CTNS exon 1 sequencing primer F	GATTGTCTACAG GGAGCTGA	This paper	Forward PCR primer for CTNS KO sequencing of genomic DNA at exon 1
CTNS exon 1 sequencing primer F	GACATGTGGTCT GAATGATTC	This paper	Forward PCR primer for CTNS KO sequencing of genomic DNA at exon 1
LAMP1 crRNA	rGrUrGrCrArCrCrAr GrGrCrUrArGrArUr ArGrUrC	Boecker *et al*, 2020	crRNA for LAMP1-GFP KnockIn tagging
CTNS crRNA	rArCrCrArGrCrUrGr ArArCrUrArGrCrAr CrCrCrArGrUrUrUr UrArGrArGrCrUrAr UrGrCrU	This paper	crRNA for CTNS-2xHA KnockIn tagging
JIP4 mutant mouse sgRNA 1	TAGTAAGGGCTA CTGTAGTG	This paper	sgRNA 1 for JIP4 KO mouse creation targeting exon 4
JIP4 mutant mouse sgRNA 2	TGGATGTGCAAA TAACGGAG	This paper	sgRNA 2 for JIP4 KO mouse creation targeting exon 4

Table S3: Summary of double stranded DNA blocks and ssDNA sequences used in this study

DNA	Sequence (5'->3')
JIP4-FLAG GenEZ ORF DNA	ATGGAGCTGGAGGACGGTGTGGTGTATCAGGAGGAGCCCGGCGGCT CCGGGGCCGTGATGTCGGAGCGGGTGTCCGGCCTGGCCGGCTCCAT CTACCGCGAGTTCGAGCGGCTTATCGGGCGCTATGACGAGGAGGTGG TCAAAGAGCTGATGCCGCTGGTGGTGGCTGTGCTGGAGAACCTGGAC TCGGTGTTCGCGCAGGACCAGGAGCACCAGGTGGAGCTGGAGCTGC TGCGGGACGACAACGAGCAGCTCATCACCCAGTACGAGCGGGAGAA GGCGCTGCGCAAGCACGCTGAGGAGAAATTCATTGAATTTGAAGACTC TCAAGAACAGGAAAAAAAGGACTTACAGACCCGAGTGGAATCTTTAGA ATCTCAAACAAGACAACTTGAGCTGAAAGCGAAAAACTATGCTGACCA GATTAGCAGACTTGAAGAAAGAGAAGCAGAACTGAAGAAGGAATATAA TGCATTACATCAAAGACACACTGAGATGATCCATAATTATATGGAACAT TTAGAAAGAACAAAACTTCATCAGCTCTCAGGGAGTGATCAACTAGAAT CCACAGCTCATAGTAGAATTAGAAAAGAACGCCCTATATCATTAGGAAT TTTCCCATTACCTGCTGGAGATGGATTGCTTACACCTGATGCTCAGAA AGGAGGAGAGACCCCTGGATCTGAGCAATGGAAATTTCAGGAATTAAG TCAACCACGTTCTCATACCAGCCTGAAGGTCAGCAATAGTCCTGAACC

bioRxiv preprint doi: https://doi.org/10.1101/2025.06.06.657909; this version posted June 9, 2025. The copyright holder for this preprint (which was not certified by peer review) is the author/funder, who has granted bioRxiv a license to display the preprint in perpetuity. It is made available under aCC-BY 4.0 International license.

	TCAGAAGGCTGTAGAACAGGAGGATGAGCTTTCTGATGTTAGCCAAGG<br>CGGATCTAAAGCTACCACTCCAGCATCAACAGCTAATTCAGATGTGGC<br>AACAATTCCTACTGATACTCCCTTAAAGGAAGAAAACGAAGGATTTGTG<br>AAGGTTACAGATGCGCCAAATAAATCAGAGATAAGCAAACACATTGAA<br>GTACAGGTAGCCCAGGAAACTAGAAATGTATCTACTGGCTCTGCTGAA<br>AATGAAGAAAAGTCAGAAGTTCAAGCAATCATCGAATCTACTCCTGAG<br>CTGGATATGGACAAAGATCTCAGTGGATATAAAGGTTCAAGCACTCCC<br>ACCAAAGGCATAGAGAACAAAGCTTTTGATCGCAATACAGAATCTCTCT<br>TTGAAGAACTGTCTTCAGCTGGCTCAGGCCTAATAGGAGATGTGGATG<br>AAGGAGCAGATTTACTAGGAATGGGTCGGGAAGTTGAGAATCTTATAT<br>TAGAAAATACACAACTGTTGGAAACCAAAAATGCTTTGAACATAGTGAA<br>GAATGATTTGATAGCAAAAGTGGATGAACTGACCTGTGAGAAAGATGT<br>GCTGCAAGGGGAATTGGAGGCTGTGAAGCAAGCCAAACTGAAACTAG<br>AGGAAAAGAACAGAGAATTGGAGGAAGAGCTTAGGAAAGCTCGGGCA<br>GAAGCTGAAGATGCAAGGCAAAAAGCAAAAGATGACGATGATAGTGAT<br>ATTCCCACAGCCCAGAGGAAACGGTTTACTAGAGTAGAAATGGCCCGT<br>GTTCTCATGGAGCGAAACCAGTATAAAGAGAGATTGATGGAGCTTCAG<br>GAAGCTGTTCGATGGACAGAGATGATTCGGGCATCACGAGAAAATCCA<br>GCCATGCAGGAAAAAAAAAGGTCAAGCATTTGGCAGTTTTTCAGCCGA<br>CTTTTCAGCTCCTCAAGTAACACGACTAAGAAGCCTGAACCACCTGTT<br>AATCTGAAGTACAATGCACCCACGTCTCATGTTACTCCGTCCGTCAAG<br>AAAAGAAGCAGCACCTTATCTCAGCTCCCTGGGGATAAGTCCAAAGCC<br>TTTGATTTCCTTAGTGAAGAAACTGAAGCTAGTTTAGCCTCACGCAGAG<br>AACAAAAGAGAGAGCAGTATCGTCAGGTAAAAGCACATGTTCAGAAGG<br>AAGACGGTAGAGTGCAGGCTTTTGGCTGGAGTCTGCCTCAGAAGTAC<br>AAACAGGTAACCAATGGTCAAGGTGAAAATAAGATGAAAAATTTACCTG<br>TGCCTGTCTATCTCAGACCTCTGGATGAAAAAGATACATCAATGAAGCT<br>GTGGTGTGCTGTTGGAGTCAATTTATCTGGTGGGAAGACCAGAGATG<br>GTGGTTCTGTTGTTGGAGCAAGTGTATTTTACAAGGATGTTGCTGGTTT<br>GGATACAGAAGGCAGTAAACAGCGAAGTGCCTCTCAGAGTAGTTTAGA<br>TAAGTTAGATCAGGAACTTAAGGAACAGCAGAAGGAGTTAAAAAATCA<br>AGAAGAATTATCCAGTCTAGTTTGGATCTGTACCAGCACTCATTCGGCT<br>ACAAAAGTTCTTATTATTGATGCTGTTCAACCTGGCAACATCCTAGACA<br>GTTTCACTGTTTGCAACTCTCATGTTCTGTGCATTGCAAGTGTGCCAG<br>GTGCACGAGAAACAGACTACCCTGCAGGAGAAGATCTTTCAGAATCTG<br>GTCAGGTAGACAAAGCATCTTTATGTGGAAGTATGACAAGCAACAGCT<br>CAGCAGAGACAGACAGCCTGTTAGGAGGCATCACAGTGGTTGGTTGT<br>TCTGCAGAAGGTGTGACGGGAGCTGCCACTTCCCCTAGTACAAATGG<br>TGCTTCTCCAGTGATGGATAAACCACCAGAAATGGAAGCAGAAAATAG<br>TGAGGTTGATGAAAATGTTCCAACAGCAGAAGAAGCAACTGAAGCTAC<br>AGAAGGGAATGCGGGGTCAGCTGAAGACACAGTGGACATCTCCCAAA<br>CTGGCGTCTACACAGAGCATGTCTTTACAGATCCTTTGGGAGTTCAGA<br>TCCCAGAAGACCTCTCCCCAGTGTATCAGTCGAGCAATGACTCAGATG<br>CATATAAAGATCAAATATCAGTACTGCCAAATGAACAAGACTTGGTGAG<br>AGAAGAAGCCCAGAAAATGAGTAGTCTTTTACCAACTATGTGGCTTGG<br>AGCTCAAAATGGCTGTTTGTATGTCCATTCATCTGTAGCCCAGTGGAG

bioRxiv preprint doi: https://doi.org/10.1101/2025.06.06.657909; this version posted June 9, 2025. The copyright holder for this preprint (which was not certified by peer review) is the author/funder, who has granted bioRxiv a license to display the preprint in perpetuity. It is made available under aCC-BY 4.0 International license.

	GAAATGTCTCCATTCCATTAAACTTAAAGATTCGATTCTCAGTATTGTAC<br>ACGTGAAGGGAATCGTGTTAGTAGCCCTGGCTGACGGCACCCTTGCA<br>ATCTTTCACAGAGGAGTGGATGGGCAGTGGGATTTGTCAAACTATCAC<br>CTCTTAGACCTTGGACGGCCTCATCATTCCATCCGTTGCATGACTGTG<br>GTACATGACAAAGTCTGGTGTGGCTATAGGAACAAAATCTATGTGGTG<br>CAGCCAAAGGCCATGAAAATAGAGAAATCTTTTGATGCACATCCCAGG<br>AAGGAGAGCCAAGTGCGACAGCTTGCGTGGGTGGGGGATGGCGTGT<br>GGGTCTCCATTCGCTTGGATTCTACGCTCCGTCTCTATCATGCACACA<br>CTTATCAACATCTACAGGATGTGGACATTGAGCCTTATGTAAGCAAAAT<br>GTTAGGTACTGGAAAACTGGGCTTCTCTTTTGTGAGAATTACAGCTCTT<br>ATGGTGTCTTGTAATCGTTTGTGGGTGGGGACAGGAAATGGTGTCATT<br>ATCTCCATCCCATTGACAGAAACAAATAAAACCTCAGGTGTACCAGGA<br>AATCGTCCTGGAAGTGTAATCCGTGTATATGGTGATGAAAACAGTGAT<br>AAAGTGACTCCAGGGACATTTATACCCTATTGTTCAATGGCACATGCA<br>CAGCTTTGCTTCCATGGGCACCGGGATGCTGTGAAATTCTTTGTGGCA<br>GTCCCAGGTCAAGTCATCAGCCCACAAAGTAGCAGTAGTGGCACGGA<br>TCTGACGGGTGACAAAGCAGGGCCATCTGCACAGGAGCCTGGTAGTC<br>AGACGCCCTTGAAGTCTATGCTTGTCATCAGTGGAGGAGAGGGCTAC<br>ATCGACTTCCGAATGGGTGATGAAGGTGGAGAATCAGAACTTCTTGGA<br>GAGGATCTTCCACTTGAACCTTCTGTCACCAAAGCAGAAAGGAGTCAC<br>TTGATAGTGTGGCAAGTGATGTATGGCAATGAGTGA
CTNS-FLAG gBlock	ATGATAAGGAATTGGCTGACTATTTTTATCCTTTTTCCCCTGAAGCTCG<br>TAGAGAAATGTGAGTCAAGCGTCAGCCTCACTGTTCCTCCTGTCGTAA<br>AGCTGGAGAACGGCAGCTCGACCAACGTCAGCCTCACCCTGCGGCCA<br>CCATTAAATGCAACCCTGGTGATCACTTTTGAAATCACATTTCGTTCCA<br>AAAATATTACTATCCTTGAGCTCCCCGATGAAGTTGTGGTGCCTCCTG<br>GAGTGACAAACTCCTCTTTTCAAGTGACATCTCAAAATGTTGGACAACT<br>TACTGTTTATCTACATGGAAATCACTCCAATCAGACCGGCCCGAGGAT<br>ACGCTTTCTTGTGATCCGCAGCAGCGCCATTAGCATCATAAACCAGGT<br>GATTGGCTGGATCTACTTTGTGGCCTGGTCCATCTCCTTCTACCCTCA<br>GGTGATCATGAATTGGAGGCGGAAAAGTGTCATTGGTCTGAGCTTCGA<br>CTTCGTGGCTCTGAACCTGACAGGCTTCGTGGCCTACAGTGTATTCAA<br>CATCGGCCTCCTCTGGGTGCCCTACATCAAGGAGCAGTTTCTCCTCAA<br>ATACCCCAACGGAGTGAACCCCGTGAACAGCAACGACGTCTTCTTCAG<br>CCTGCACGCGGTTGTCCTCACGCTGATCATCATCGTGCAGTGCTGCCT<br>GTATGAGCGCGGTGGCCAGCGCGTGTCCTGGCCTGCCATCGGCTTCC<br>TGGTGCTCGCGTGGCTCTTCGCATTTGTCACCATGATCGTGGCTGCAG<br>TGGGAGTGATCACGTGGCTGCAGTTTCTCTTCTGCTTCTCCTACATCA<br>AGCTCGCAGTCACGCTGGTCAAGTATTTTCCACAGGCCTACATGAACT<br>TTTACTACAAAAGCACTGAGGGCTGGAGCATTGGCAACGTGCTCCTGG<br>ACTTCACCGGGGGCAGCTTCAGCCTCCTGCAGATGTTCCTCCAGTCCT<br>ACAACAACGACCAGTGGACGCTGATCTTCGGAGACCCAACCAAGTTTG<br>GACTCGGGGTCTTCTCCATCGTCTTCGACGTCGTCTTCTTCATCCAGC<br>ACTTCTGTTTGTACAGAAAGAGACCGGGGTATGACCAGCTGAACGATT<br>ATAAAGATGATGATGATAAATAG

bioRxiv preprint doi: https://doi.org/10.1101/2025.06.06.657909; this version posted June 9, 2025. The copyright holder for this preprint (which was not certified by peer review) is the author/funder, who has granted bioRxiv a license to display the preprint in perpetuity. It is made available under aCC-BY 4.0 International license.

CTNS-RUSH gBlock (SBP in lowercase)	ATGATAAGGAATTGGCTGACTATTTTTATCCTTTTTCCCCTGAAGCTCG<br>TAGAGAAATGTGAGTCAAGCGTCAGCCTCACTGTTCCTCCTGTCGTAA<br>AGCTGGAGAACGGCAGCTCGACCAACGTCAGCCTCACCCTGCGGCCA<br>CCATTAAATGCAACCCTGGTGATCACTTTTGAAATCACATTTCGTTCCA<br>AAAATATTACTATCCTTGAGCTCCCCGATGAAGTTGTGGTGCCTCCTG<br>GAGTGACAAACTCCTCTTTTCAAGTGACATCTCAAAATGTTGGACAACT<br>TACTGTTTATCTACATGGAAATCACTCCAATCAGACCGGCCCGAGGAT<br>ACGCTTTCTTGTGATCCGCgacgagaagaccactggttggcgaggtggacacgttgttga<br>aggactggctggggaacttgaacaacttcgtgcacgactggagcatcacccacaaggtcaacgtgaa<br>ccaAGCAGCGCCATTAGCATCATAAACCAGGTGATTGGCTGGATCTACT<br>TTGTGGCCTGGTCCATCTCCTTCTACCCTCAGGTGATCATGAATTGGA<br>GGCGGAAAAGTGTCATTGGTCTGAGCTTCGACTTCGTGGCTCTGAACC<br>TGACAGGCTTCGTGGCCTACAGTGTATTCAACATCGGCCTCCTCTGGG<br>TGCCCTACATCAAGGAGCAGTTTCTCCTCAAATACCCCAACGGAGTGA<br>ACCCCGTGAACAGCAACGACGTCTTCTTCAGCCTGCACGCGGTTGTC<br>CTCACGCTGATCATCATCGTGCAGTGCTGCCTGTATGAGCGCGGTGG<br>CCAGCGCGTGTCCTGGCCTGCCATCGGCTTCCTGGTGCTCGCGTGGC<br>TCTTCGCATTTGTCACCATGATCGTGGCTGCAGTGGGAGTGATCACGT<br>GGCTGCAGTTTCTCTTCTGCTTCTCCTACATCAAGCTCGCAGTCACGC<br>TGGTCAAGTATTTTCCACAGGCCTACATGAACTTTTACTACAAAAGCAC<br>TGAGGGCTGGAGCATTGGCAACGTGCTCCTGGACTTCACCGGGGGCA<br>GCTTCAGCCTCCTGCAGATGTTCCTCCAGTCCTACAACAACGACCAGT<br>GGACGCTGATCTTCGGAGACCCAACCAAGTTTGGACTCGGGGTCTTC<br>TCCATCGTCTTCGACGTCGTCTTCTTCATCCAGCACTTCTGTTTGTACA<br>GAAAGAGACCGGGGTATGACCAGCTGAACGATTATAAAGATGATGATG<br>ATAAATAG
CTNS-2xHA Alt-R HDR Donor Oligo (ssDNA repair template)	CAGGGCCCAACCGCTTCTCCGGCCTTCGCTGGGTGAGGCCTTCCCCA<br>GCAGGGCACGAGGCCAGAGGCTGGGTACACTGGGTCCTTGGGTGCT<br>ATGCATAGTCCCGGGACGTCATAGGGATAAGCGTAATCTGGAACATCG<br>TATGGGTAGTTCAGCTGGTCATACCCCGGTCTCTTTCTGTA

Table S4: Summary of siRNAs used in this study

siRNA	Supplier	Reference
Dharmacon ON-TARGETplus Non-Targeting siRNA Pool	Horizon Biosciences	D-001810-10-05
Dharmacon OnTarget Plus siRNA smartPOOL TMEM55A	Horizon Biosciences	L-013808-01-0005
Dharmacon OnTarget Plus siRNA smartPOOL TMEM55B	Horizon Biosciences	L-016425-02-0005
